# Targeting Oxidative Stress for Disease Prevention and Therapy: Where Do We Stand, and Where Do We Go from Here

**DOI:** 10.3390/molecules25112653

**Published:** 2020-06-07

**Authors:** Cristina Vassalle, Maristella Maltinti, Laura Sabatino

**Affiliations:** 1 Fondazione CNR-Regione Toscana G Monasterio, 56124 Pisa, Italy; maristella@ftgm.it; 2 Institute of Clinical Physiology, National Research Council, 56124 Pisa, Italy; laura.sabatino@ifc.cnr.it

**Keywords:** oxidative stress, blood, pharmacological target, antioxidant supplementation, disease prevention, disease therapy, miRNAs

## Abstract

Oxidative stress (OxS) is one of the main processes related to aging and a common denominator of many different chronic/degenerative diseases (e.g., cardiovascular and neurodegenerative conditions and cancer). Thus, its potential modulation by supplementation/pharmacological therapy caused a lot of interest. However, these expectations have been mitigated by the obtainment of controversial results (beneficial, null, or adverse effects) following antioxidant interventions. Here, we discuss the current understanding of OxS assessment in health and disease, challenges and the potential of its evaluation in clinical practice, and available and future development for supplementation and pharmacologic strategies targeting OxS.

## 1. Introduction

A low production of reactive oxygen species (ROS) is normally continuous, being essential in the regulation of many different biological processes. Thus, the alteration of the oxidative stress (OxS) status in its oxidant/antioxidant components has been identified as one of the most important determinants underlying aging-related processes, and onset and progression of chronic/degenerative diseases (e.g., atherosclerosis, neurodegenerative disease, cancer) [1,2]. However, the delicate balance of oxidants and antioxidants must be carefully evaluated. The ROS excessive production is the most ascertain way by which this damage occurs [3]. In particular, cellular events (mitochondrial activity, inflammatory processes, and several enzymatic complexes), as well as lifestyle habits, environmental pollution, radiation, smoking, and some drugs may contribute to an OxS surplus, which in turn may damage critical biomolecules, including lipids, proteins, and nucleic acids [3]. Furthermore, a critical role in the oxidative stress response is played by Keap1/Nrf2 and NFκB/IκB pathways, which modulate the transcription of many antioxidant and detoxification genes [4,5]. Specifically, Nrf2 induces the expression of detoxification and antioxidant enzymes under the oxidative stress, where Keap1 translocates Nrf2 from the cytoplasm to the nucleus. In the nucleus Nrf2, the antioxidant response element (ARE) connection activates endogenous antioxidant and detoxificant responses [5]. Moreover, non-radical species such as hydrogen peroxide (H_2_O_2_) or singlet molecular oxygen are responsible for fine-tuning physiological redox signaling, acting as second messengers [4].

An important concept to keep in mind when evaluating the oxidative stress status, is that whether an elevated oxidant status induces biomolecule injury, the maintenance of a physiological level of oxidative stress is crucial to molecular and cellular processes through redox signaling [4]. The problem is to define this required eustress (physiological oxidative stress). In fact, the combinations by which redox signaling integrates are decisively complex. It is not sure that a decrease in the oxidizing component is always positive, nor that an increase in the antioxidant counterpart is always positive. Accordingly, an increase of oxidants does not necessarily lead to the OxS status when counterbalanced by an antioxidant increase, as well as an antioxidant decrease does not necessarily turn out to be unfavorable if it happens in parallel to the oxidant reduction [6]. Conversely, as the production of low levels of oxidant species is essential to allow for an upregulation of endogenous antioxidant defenses, their prolonged, excessive reduction may be even detrimental [7]. In the same way, a too high antioxidant concentration might be adverse, as they can paradoxically favor the generation of oxidative species (e.g., uric acid) [6]. Accordingly, whether the Nrf2/Keap1 system is protective, its overactivation can be adverse, as in cancer and in resistance to chemotherapy [8]. Therefore, it is controversial how the activation, or alternatively the inhibition, of the Nrf2 system may be efficiently used in the prevention or treatment of cancer or other diseases. 

Here, we discuss the main topics related to OxS evaluation in health and disease, OxS biomarkers and assay limits and advantages, interpretation issues, actual results, and future development in supplementation and pharmacologic intervention targeting OxS. 

## 2. Oxidative Stress Biomarkers: Which One to Use?

**Figure 1:**
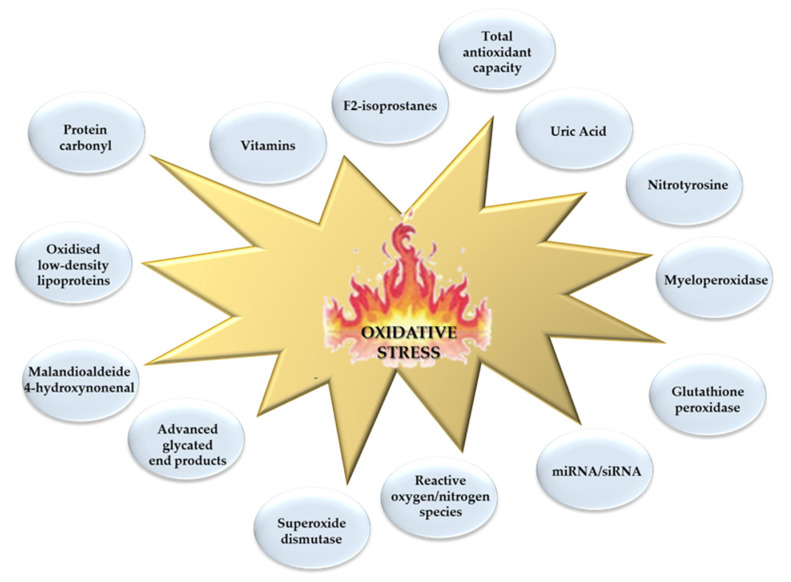
Main commonly measured biomarkers related to the oxidative stress multi-entity.

Under the term “oxidative stress” many molecular events and cellular processes are included, which reflect a great variety of pathways [9]. In the attempt to determine this OxS dynamic multiplicity, the number of OxS biomarkers measurable in a biological sample (generally in blood, but also in urine and saliva), and belonging to both the oxidant and antioxidant counterpart (lipids, proteins, nucleic acids, and antioxidants enzymatic and nonenzymatic antioxidants), is exponentially increased in the course of time [9] (Figure 1).

In particular, the most widespread biomarkers include those pertaining to lipid peroxidation (e.g., malondialdehyde (MDA) or thiobarbituric acid reactive substances (TBARS), isoprostanes (IsoP), and hydroperoxides (LOOH)). Among antioxidant enzymes, superoxide dismutase (SOD) or glutathione peroxidase (GPx) are commonly measured, as well as vitamins or uric acid to assess the nonenzymatic antioxidant capacity.

However, in certain conditions behind single antioxidant biomarkers, which likely mirror only specific aspects of the whole scenario, and thus are partially informative, it may be possible to evaluate the total antioxidant contribution, by using the total antioxidant capacity (TAC), the ferric reducing antioxidant potential (FRAP), the total radical-trapping antioxidant parameter (TRAP), and the oxygen radical antioxidant (ORAC) assays [6].

Moreover, to describe as accurately as possible the multifaceted OxS entity, several indices, which generally include both the oxidant and the antioxidant counterparts, have been proposed and recently reviewed (e.g., oxidative stress index (OSI), and oxidative-index (OXY-index), thiol ratios (SH/TT, SS/-SH, and SS/TT), glutathione ratio (GSSG/GSH), oxidative stress score (OSS)) [10]. Generally, these indices include in their formula at least one component of the oxidant and antioxidant counterpart, until the so-called “oxidative stress profile,” where the combination of about 50 biomarkers were proposed to assess the oxidative status [11,12]. This strategy may be promising, especially when biomarkers with a low degree of correlation are selected, thus considering different aspects of the OxS multi-entity. However, this procedure increases the costs of evaluation, and renders a feasibility and interpretation complex. Moreover, as these indices consider different biomarkers in their formulas, results obtained in different conditions may diverge.

In general, the choice of a specific biomarker depends on the improvement of the medical approach after the test (post-test probability) compared to the one that would occur without testing (pretest probability). In this context, the addition of OxS-related biomarkers to a general panel may be a further alternative possibility to improve the performance to established models of risk prediction including, for example, anthropometric, clinical, instrumental, and other biochemical markers [13,14]. Effectively, first attempts have proven that the addition of OxS-related biomarkers to existing panels is able to increase the prediction of cardiovascular events in different cardiovascular patient cohorts [15,16,17].

Nonetheless, the importance of an OxS biomarker may greatly vary in different clinical and experimental contexts. Thus, the incremental value of different redox-related biomarkers according to clinical settings (screening, diagnosis, prognosis, monitoring, and treatment), pathophysiological conditions (disease risk or disease presence or stage), specific population (general population, patients) remains to be carefully evaluated [9].

## 3. Oxidative Stress Assessment: Limits and Challenges

One main advantage (but also disadvantage) to assess the OxS status is the variety of methodologies and assays available to evaluate the great number of biomarkers in different biological samples. Each method retains different advantages and disadvantages, sensibility, practicability, and costs [6].

Moreover, many issues associated to the preanalytical and postanalytical phases may contribute to complicate the interpretation of results and need to be further standardized.

### 3.1. Preanalytical Issues

“Preanalytical factors” are all the variables that may influence a sample prior to analysis in the laboratory. The sources of preanalytical variability can be summarized into four main categories:

sample collection (e.g., biological sample type, collection tube anticoagulant, daytime of withdrawal),
sample handling (e.g., temperature, time from collection to laboratory delivery, delay before assaying),
sample processing (e.g., centrifugation, hemolyzed samples),
sample short- and long-storage (e.g., time and temperature) and stability (e.g., freeze-thaw cycles).

Moreover, diurnal variations, fasting status, subjects’ posture (supine vs. sitting vs. standing) may be considered as determinants of the preanalytical phase.

Regarding the sample type, and in particular for saliva, an increase of secretion by food ingestion, presence of carriers, use of chewing-gum mastication or citric acid stimulation, as well as possible blood contamination, must be considered [18]. For urine, the 24-h collection may introduce further difficulties related to a correct sample conservation during this time in order to ensure the adequate stability of analytes. Although practicable in urine and saliva, the most diffuse specimen to assess the OxS status remains blood, which is the main focus of this review. For what concerns the anticoagulant in blood samples, the anticoagulant ethylenediaminetetraacetic acid (EDTA) appears to be less preferable, as iron chelation may stabilize the reducing capacity of ferrous iron [19]. The circadian rhythm must be considered for some analytes, as well as diet interference, and for these reasons, a withdrawal protocol characterized by the fasting status in the morning is preferable for all samples.

All samples should be delivered to the laboratory in the shortest possible time, the cold chain must not be interrupted after collection. A prompt refrigerated centrifugation appears appropriate, followed by immediate assaying, or storage at a low temperature (−20, −80 °C) to avoid sample autoxidation and assure stability over time. With these precautions, many OxS-related biomarkers showed a very good degree of stability in a sample stored up to one year at −20 and −80 °C [20,21].

Moreover, hemolysis and a high lipid content in samples may represent important preanalytical concerns, and samples presenting marked signs of these interferences should not be tested. The sample pretreatment (e.g., for the case of MDA and other OxS biomarkers, the use of stabilizers such as butylated hydroxytoluene (BTH)) can be adopted to improve evaluation. Repeated freeze-thaw cycles should be avoided.

### 3.2. Analytical Issues

The main analytical issue concerns the agreement on which method to use [22]. Many different methods are currently available, focusing on different biomarkers related to the OxS multi-entity, have different sensibilities and specificities, and each one retains advantages and disadvantages.

To assess the oxidative damage through lipid peroxidation, which is the most commonly used approach, MDA, 4-hydroxynonenal (HNE), and 8-*iso*-Prostaglandin F2-alpha (*8*-*iso* PGF2α, an isoprostane produced by the nonenzymatic peroxidation of arachidonic acid in membrane phospholipids) are generally used [6]. In this context, the great availability of results evidenced the importance of these biomarkers for evaluation of the OxS status in chronic/degenerative diseases [6,23,24,25].

In particular, the widespread of MDA measurement through the TBARS (spectrophotometric or fluorimetric analysis) is related to its ease-of-use and cheapness that favor its applicability to large numbers of samples [6]. Notably, this assay retains a low specificity (many other chemically reactive carbonyl groups-containing compounds may react with TBA and interfere with the MDA evaluation), with respect to other more robust technologies (such as mass spectrometry (MS), singly or tandem (MS/MS) such as gas chromatography-mass spectrometry (GC-MS and GC-MS/MS), liquid chromatography-mass spectrometry (LC-MS and LC-MS/MS)) [6].

Due to the low specificity of TBARS reactions, results obtained by different methods/assays may not be comparable. Nonetheless, the TBARS assay specificity can be improved by adduct separation phases, for example by using HPLC [26,27]. Moreover, the majority of the methods were used to assess MDA levels, employ derivatization reagents, which react with MDA carbonyl groups (e.g., 2,4-dinitrophenylhydrazine in HPLC, pentafluorophenyl hydrazine in GC and GC-MS, and 3-nitrophenyl hydrazine in LC-MS/MS) [28,29,30].

Furthermore, for isoprostanes, GC-MS and LC-MS/MS remain the best methods for their assessment, although expensive and requiring specialized instrumentations and operators. Thus, the use of enzyme-linked immunosorbent (ELISA) kits for the assessment of F2-IsoPs is very diffuse, even if more inaccurate and unspecific [6,31]. The ELISA is based on an immune antigen-antibody reaction in a competitive binding test. As antibodies recognize specific antigens, only certain metabolites can be measured by the assay, with great discrepancies from results obtained by using GC-MS [6]. Moreover, ELISA may overestimate the concentration, as the antibodies used in ELISA may cross-react with other metabolites, whereas GC/MS appears more selective [6].

For the antioxidant counterpart, it is possible to evaluate a single antioxidant, or estimate the total antioxidant capacity [6]. However, as there is a great number of molecules and pathways involved, an antioxidant individual quantification may give only a partial point of view of the entire scenario. Thus, it could appear reasonable to evaluate more antioxidants through different tests. Nonetheless, the parallel assessment of many antioxidants in a sample may be excessively expensive and time consuming, requiring many different instruments and tests. Moreover, interferences and synergic/antagonist interactions can be lost by a single evaluation approach. Alternatively, the total antioxidant capacity can be assessed. However, also in this case, the use of different methods, may render results hardly comparable, because each one is based on a different principle, as well as the contribution of a single antioxidant to the final results may be variable for each test [6].

Many assays can be performed at a fixed time or with a kinetic trend, which did not give the same results. Paradoxical prooxidant activities of antioxidants in certain conditions (according to microenvironment and concentration), may be also considered in the interpretation of results [6]. Consequently, there might be a low agreement between different total antioxidant capacity methods, and different correlations between total antioxidant capacity assays with other oxidative tests.

### 3.3. Postanalytical Issues

For the postanalytical phase, a major issue is represented by the lack of a shared agreement on reliable reference values (at least cut-off). In this context, we may face two possibilities: 1) Screening: Availability of a single value versus 2) Monitoring: Availability of serial measurements for the same patient. In the evaluation of a single value, the main problem is to have a threshold value, to possibly classify the biomarker as positive or negative with respect to that given cut-off, or, even better, differentiate oxidative eustress by an oxidative distress (excessive and toxic oxidative burden) [4].

Unfortunately, at the moment, it has not been clearly established as a recognized cut-off value for none of the OxS biomarkers available [9].

In the second case, the dynamic variation of the biomarker over time must be interpreted (e.g., postinterventional variation, follow-up). In this context, significant changes in the value of the biomarker may indicate a greater OxS.

Importantly, in the interpretation of results, the observed value may be influenced by many factors, such as genetic (e.g., familiarity), physiological (e.g., age, gender, environmental factors, diet, pregnancy), lifestyle habits (physical activity, smoking habit, alcohol abuse, stress, anxiety, drugs), intra- and inter-subject variability (biological variability), and circadian rhythm (fluctuations in the values of some analytes during the day, week, month/season).

The use of different measure units may challenge the interpretation of results, causing confusion and potentially leading to misclassification. Moreover, the biomarker circulating value is affected by different determinants, such as distribution volume, or its metabolism and clearance that can be carefully considered.

## 4. Antioxidant Supplementation: Challenges in the Interpretation of Results

As the role of OxS in the pathogenesis of aging processes and many chronic degenerative diseases is undoubted, several attempts have been conducted to investigate possible beneficial effects of the antioxidant therapy. The rationale of exogenous supplementation is to maintain wellbeing and health in the general population, preventing the development of a diseased status, worsening and complications in patients. However, to date, the majority of epidemiological studies did not confirm any evidence of antioxidant supplementation proven benefits, especially in the cardiovascular field [32,33,34,35]. It is also important to remind that the interpretation of available results is effectively complex for many reasons, including not only the choice of the dose, the type of antioxidant, the single versus multiple approach, the time of supplementation, the population suitable to be treated, but also all the difficulties we have just discussed in the field of OxS, related to the fact that the methods often diverge.

Whether many results indicate that this supplementation may be useful in the primary and secondary prevention of chronic diseases, other trials involving a great number of subjects have not confirmed this hypothesis and available results are often contradictory. These controversial findings and difficulties in the interpretation may be due to several factors.

As we previously discussed, one issue is the choice of the biomarker/assay/method used to evaluate OxS, as each one only partly reflects the entire oxidative scenario, and none has been recognized as the “gold standard” for the clinical setting.

In the cardiovascular field, the main utilized molecules are vitamin A, ascorbic acid (vitamin C), α-tocopherol (vitamin E), folic acid, niacin, β-carotene, selenium, zinc, alone or in combination, or in a multivitamin/mineral mix with mixed (beneficial/null/adverse) results [34]. However, in addition to the more experimented antioxidants (e.g., vitamins C and E), other untested antioxidant molecules could exist, even potentially more effective than those used so far.

In addition, the efficacy of antioxidants in neurodegenerative diseases is doubtful. In this context, vitamins E, C, and B12 have been largely studied as a potential effective intervention for Alzheimer’s disease [36].

As an example, for vitamin E, experimental models testify that supplementation by itself or in combination with other antioxidants or anti-inflammatory compounds, may be a good tool to improve cognitive and memory deficits through improvement of the OxS status [37]. However, available clinical evidence remains conflicting and, as such, inconclusive [38].

In fact, a large number of confounders must be taken into account in the interpretation of results, for example, the dose, type, and/or antioxidant combinations [39]. The effects of other antioxidants derived by diet, may potentially interfere (e.g., interactions/synergisms between antioxidants) rendering the interpretation of results more difficult. Other factors, such as aging, smoking habit, radiation exposure, low physical activity, and some drugs may also increase the need of vitamin intake [40]. It is also necessary to identify and stratify populations to target subjects that are in a state of OxS and benefit more from antioxidant supplementation.

Dosage is an important variable that needs to be carefully evaluated. For example, for vitamin E, the most effective results were obtained using higher doses [38]. Thus, in some cases, vitamin E may not have been high enough to obtain a sufficient blood concentration for the perceivable benefit [38]. In fact, it has been estimated that the supplementation that must allow a threshold value to reach beneficial effects can be expressed (in the case of vitamin E, this value has been estimated at 800 IU/d) [41]. Conversely, although high doses of vitamin E do not seem to be associated with increased death and side effects, a too high dosage might induce adverse effects, analogously to other antioxidants, which may act as pro-oxidants in certain conditions, worsening the OxS status [42]. In any case, to avoid this possibility, one option could be the monitoring of plasma vitamin E or other antioxidants at baseline and during the supplementation, which at the moment is not done for almost all the studies. Another issue that should be addressed is the timing of study initiation. In fact, an early administration might be more useful in reversing mild damage, instead resulting in being ineffective when the injury is well established. In this context, the severity of the disease, cardiovascular or neurological, may be important to determine the success of the intervention.

In the cancer field, many different studies confirmed the benefits of antioxidant supplementation in large scale clinical trials [32,43]. It is well clear that the OxS may contribute to several phases of cancer promotion and progression (e.g., proliferation, survival, and resistance) [41]. However, ROS generation cannot be considered universally dangerous. In fact, continuous low levels of ROS production have been demonstrated as a necessary stimulus for upregulation of endogenous antioxidants [7]. Moreover, the complex redox interconnection is based on an accurate fine-tuning balance between ROS production and scavenging, where a highly reducing state may be potentially harmful too [44]. Thus, the maintenance and recovery of redox homeostasis, although difficult to determine, would be the target of a new treatment concept emerging in the oncologic field.

Interestingly, some data suggest that the inhibition of antioxidant defense and increased ROS are able to trigger programmed cancer cell death [32]. Accordingly, a number of anticancer drugs that are able to increase ROS generation through modulation of specific ROS-generating pathways in different cancer types are under study [32]. In this scenario, mechanistic OxS-related biomarkers can help to identify patient subgroups, which differently respond to specific pharmacological strategies. Thus, it will be important to target specific critical redox pathways and increase the selectivity of these anticancer OxS-related pharmacological approaches. Clearly, the identification of specific sources of OxS to target remains very challenging, due to the very different and dynamic nature of the pathophysiological mechanisms underlying redox homeostasis. In any case, this approach appears promising and may represent an alternative/additive tool to the traditional strategy of targeting oncogenes and tumor suppressor genes [32].

The Nrf2/Keap1 pathways, a key cellular defense mechanism determinant in the modulation of a variety of genes (not only those belonging to the antioxidant system, but also genes that control a number of processes including immune and inflammatory responses, fibrosis, and carcinogenesis), has attracted attention as a possible therapeutic target for the prevention or treatment of diseases [45]. The main mechanisms by which activators may increase Nrf2 expression, and its translocation into the nucleus, where it starts its transcriptional effects and induces the antioxidant response, include: (a) Activation of upstream kinases, which favor the release of Nrf2 from Keap1; (b) modification of Keap1 cysteine residues, which favors Nrf2 dissociation; (c) block of Nrf2 ubiquitination and/or proteasomal degradation [46]. Several activators of Nrf2 have been identified, some plant-derived (e.g., resveratrol, curcumin), others synthetic compounds (e.g., dimethyl fumarate). However, as overactivation of the Nrf2 pathway has been found in many cancer cellular lines and human cancer tissues, a cautious and vigilant approach in the use of Nrf2 activators must be kept, which require further deepening [45].

## 5. Cardiovascular Disease Drugs with Antioxidant Properties as an Example for Shared Disease Pathways and Common Pharmacological Prevention

Many common cardiovascular drugs, such as statins, β-blockers, angiotensin-converting enzyme inhibitors, and angiotensin receptor blockers exhibit antioxidant effects.

The same agents retain a role in cancer and neurodegenerative conditions suggesting that targeting common (in this case OxS-related) pathways may represent a successful pharmacological strategy for the prevention of multiple diseases, although always keeping in mind that the OxS damage does not represent the only event in the pathogenesis of these conditions [1,47].

For example, statins, although with different characteristics depending on their mechanism of action, renal excretion, and pharmacological activities, show antioxidant properties and anti-inflammatory effects (e.g., cytokine reduction). In particular, at a cardiovascular level, the main antioxidant effects of statins are thought to be mediated by inhibition of the small proteins Rho and Rac [48,49,50]. In fact, statin-related inhibition of hydroxymethylglutaryl coenzyme A reductase prevents the production of mevalonic acid, which is a precursor of nonsteroidal isoprenoids (lipid attachment molecules for Rho and Rac). In particular, Rho negatively regulates endothelial nitric oxide synthase, whereas Rac serves as a key factor for the assembly and function of NADPH oxidase (a major source of ROS) and superoxide production [48,49,50]. Moreover, statins may also target other NADPH oxidase subunits (reduce expression of p22phox and NOX1, prevent isoprenylation of p21), reducing enzyme activity [48,49,50]. These mechanisms are emerging as important factors in cancer and neurodegeneration, where the use of statins appear rational [51,52,53].

## 6. Towards Genetic-Based Approaches to Target Oxidative Stress: Endogenous Perspective and Epigenetic MiRNA-Based Therapeutic Response

### 6.1. Endogenous Stress: Genome Instability, Antioxidants vs. Oxidation Therapy

Endogenous stress represents a major source of genome instability. It is true that genetic variability is required in physiological processes, being the driving force for genomic evolution. However, as it is fundamental to maintain a perfect balance between diversity and stability, many intracellular mechanisms are involved in the regulatory machine in order to protect and prevent the cell from deleterious genetic modifications, and preserve genomic stability [54]. Thus, as ROS are the main source of genetic damage, it is reasonable that the most updated therapeutic approaches aim to counterbalance ROS production inside the compromised cell. However, the high cytotoxic nature of ROS can be also used to selectively kill cancer cells, by the so called “oxidation therapy” [32]. Moreover, the line between benefit and damage strictly depends on the strength of OxS. Intense OxS induces the activation of transcriptional factor kappa B (NFkB) and activates an inflammatory response and tissue damage through prostaglandin and cytokine production [55]. NFkB is considered a “switch”, whose activation triggers one of the most valuable gene expression regulations [56]. Ultraviolet radiation, cigarette smoke, ozone, and many other stimuli activate NFkB which is mediated through the production of ROS. Oxidants can be counteracted by antioxidants that modulate NFkB activation. NFkB is normally present in the cytoplasm of unstimulated cells, as a transcriptionally potentially active dimer bound to an inhibitor protein called IkB [57]. Upon stimulation by an increased intracellular ROS such as H_2_O_2_, superoxide (O_2_^−^), or hydroxyl radical (·OH), IkB is rapidly phosphorilated and the NFkB dimer is released and activated [57]. In particular, H_2_O_2_ has been proposed as a central second messenger to NFkB activation [58,59]. Instead, when a moderate OxS occurs, the nuclear factor-erythroid 2-related factor 2 (Nrf2) is activated and induces the transcription of antioxidant response elements (ARE), a cis-acting element involved in the transcriptional regulation of antioxidant gene products, increasing the endogenous antioxidant capacity of response, such as SOD, GPx, glutathione-*S*-transferase (GSTs), catalase (CAT), heme-oxygenase-1 (HO-1), NADPH-quinoneoxidoreductase (NQO-1), and heat shock proteins (HSP) [55,60]. The cytoplasmic protein Kelch-like ECH-associated protein (Keap1) interacts with Nrf2 and represses its function. Therefore, the Keap1-Nrf2-ARE signaling pathway is one of the most investigated responses of the cell to oxidative stress, inflammation, and carcinogenesis [5,61]. A possible strategy, aiming to the individuation of a therapeutic agent in several diseases involving oxidative stress conditions, may pass through targeting this specific pathway. So far, some clinical trials have involved a few agents known as an activator of the Nrf2/ARE pathway, e.g., for the treatment of prostate cancer [62], and diabetes mellitus [63].

### 6.2. Epigenetic MiRNA-Based Approach

Epigenetics have been reported to be profoundly involved in OxS responses and epigenetic mechanisms are emerging over the last years as potentially useful tools to design therapeutic approaches.

In many different pathologies, such as cancer, diabetes mellitus, cardiovascular, neurodegenerative and rheumatic diseases, ROS-related signaling pathways prompt transcriptional and epigenetic dysregulation, inducing chronic low-grade inflammation, platelet activation, and endothelial dysfunction.

Epigenetic mechanisms, consisting of heritable changes in gene expression with no change in the DNA sequence, mainly include micro-RNA (miRNA) regulation, DNA methylation, and histone modification [64,65].

In particular, we aim to focus on relations between OxS and miRNA regulation and to point out new diagnostic and therapeutic perspectives in the use of miRNAs as potential mediators of OxS. MiRNAs derive from noncoding portions of genome, acting in RNA silencing and posttranscriptional regulation of gene expression. As the attested biological significance of this class of small RNAs was increasing, several efforts have been done to characterize their role in different diseases. OxS can modulate (increase or inhibit) the expression level of specific miRNAs, as observed, for example, with sirtuins and miR-34a in bronchial epithelial cells [66] or with p53 and let-7 family in several tumor cells [67,68,69]. Many studies attest the role of noncoding miRNAs in the posttranscriptional regulation of cellular metabolism during inflammation, cell proliferation, angiogenesis, and apoptosis [70].

Over the past decades, numerous miRNAs have been studied as sensitive and specific biomarkers for the diagnosis and monitoring of human diseases, and as potential therapeutic targets [71]. Current strategies are based on the expression or inhibition of specific miRNAs and require the delivering of specific synthetic oligoribonucleotides (ORNs) that mimic the original miRNAs (miR-mimetic), in case an overexpression is necessary, or acting as a single-stranded antisense (called anti-miR), aiming to sequester the original miRNA and proceed to its inhibition [72]. In this approach some problems may occur, such as the issues related to RNA stability of ORNs and the delivery barriers, which have to be further evaluated.

**Figure 2:**
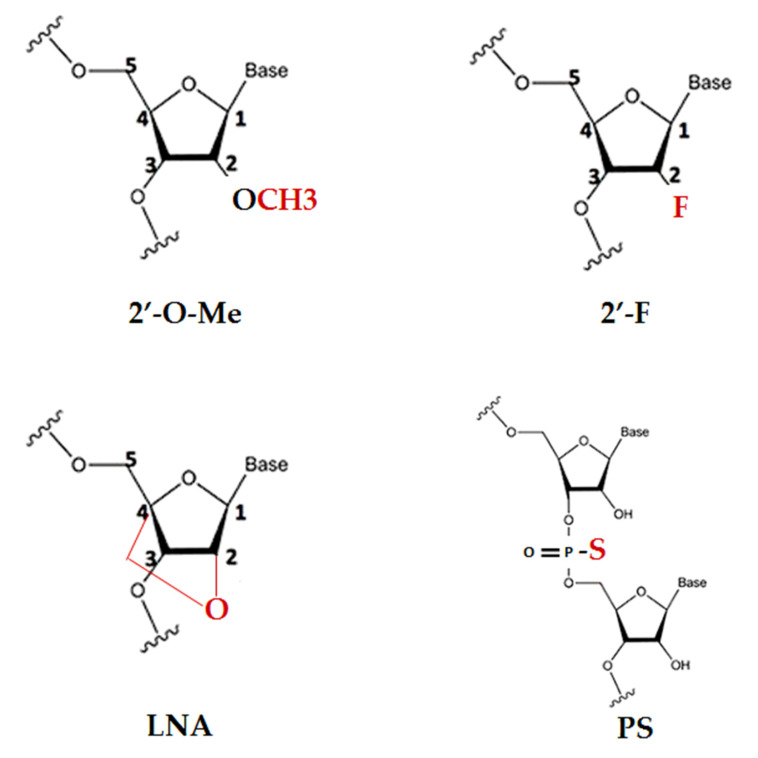
Examples of chemical modifications of nucleic acid analogs: 2′-substitutions (2′-*O*-methyl, and 2′-fluoro); locked nucleic acid modification (LNA); 5′-phosphorothioate (PS).

RNA is unstable in vivo, due to ribonucleases in the serum and in cells, therefore, chemical modifications are required to enhance their properties without compromise to their activity. Main modifications regard the typical nucleic acid ribose sugar backbone with 2′-modifications (2′-*O*-methyl (2′-OMe); locked nucleic acid (LNA); 2′-fluor (2′-F); phosphorothioate (PS)), which tend to accumulate in a characteristic pattern, mostly in the liver, kidney, and phagocytically active cell types, are a possible strategy to achieve this goal (Figure 2) [73]. However, it is important to consider that each chemical modification or length change in a possible anti-miR may determine deviations from the original pattern. This aspect has to be deeply explored at an early stage of preclinical evaluation.

Thus, the pharmacokinetic behavior of anti-miR or miR-mimetic has to be further investigated. While anti-miR or miR-mimetic are cleared from the plasma within hours, their half-lives in the tissues are much longer, even weeks, and this might provide more durable beneficial effects even when the concentration in plasma results is absent. In clinical trials, since it is not always possible to obtain tissue samples to measure drug levels, it is crucial to define reliable tissue surrogates, such as peripheral blood monocytes, or subcutaneous fat biopsies to test drug levels [72].

Human diseases are generally very complex, and the deregulation of multiple genes is often the crucial step in the transformation of normal into pathological cell. Thus, the modulation of a relatively small number of miRNAs may amplify protective effects in the redox balance, influencing hundreds of transcripts, and making the crosstalk between different cellular pathways possible [74]. In this optic, the multi-targeting nature of miRNAs renders them very suitable as a therapeutic tool [75]. In fact, differently from other approaches, a single anti-miR drug might affect families of genes under the control of the target miRNA. On the other hand, this miRNA spectrum of influence may hint to a serious risk if the bond to unexpected targets occurs, producing undesired changes in gene expression [75]. Furthermore, redundant cellular pathways can limit the efficiency of monogenic targeting compounds [76]. Thus, in miRNA-targeting or mimicking therapeutics, two aspects have to be considered, one related to the chemistry-related effects and one due to the consequences of the interaction with unintended target RNAs (off-target effect) [72].

**Figure 3:**
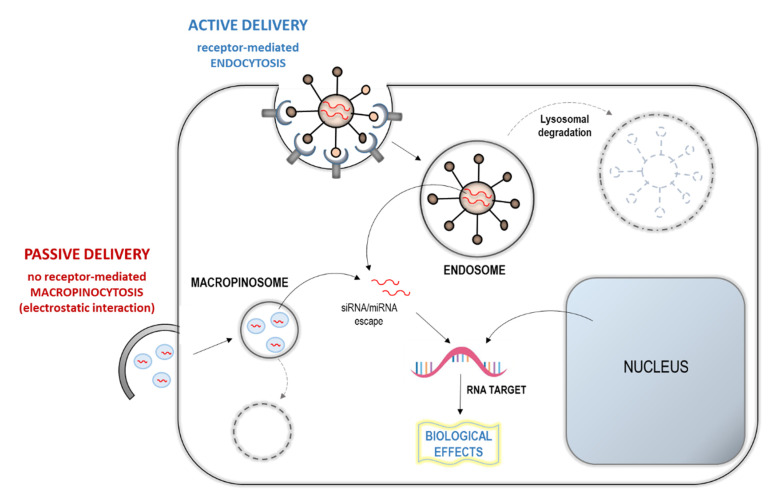
Micro-RNA/small interference-RNA (miRNA/siRNA) delivery strategies: (1) Active: By receptor-mediated endocytosis (as in the figure), or conjugated to cholesterol, aptamers, membrane-penetrating polymers, etc. and (2) passive: By no receptor-mediated micropinocytosis of miRNA/siRNA-carrying nanoparticles, liposomes-like particles, etc. by electrostatic interaction with a cellular membrane.

In a miRNA-based therapy, another major challenge is the delivery of these RNAs to the target site of action. A fundamental step in the therapeutic application is the correct delivery of the drug to the targeted organs, in order to obtain the optimal treatment specificity [77]. Considering the intravenous injection approach, the delivery strategies can be divided into passive or active (Figure 3) [73,78]. The passive strategies take advantage of the capability of organs such as the liver, the spleen, and the lymph nodes to internalize the accumulated particles.

On these bases, specifically designed nanoparticles or liposome-like particles incorporating RNAs can be used to target these organs. Differently, the active strategies combine the RNA with a specific carrier that will bind to the cells of interest and will enter the cells by endocytosis [73,78]. It is conceivable that these structural and delivery approaches, even though highly efficient, further complicate the development of miRNA therapeutics. In fact, each approach for delivering has its own merits and shortcomings in the target organism. The optimal delivery strategy may vary depending on the target tissue and cell type and the disease that is treated.

To advance an anti-miR drug in a clinical context, a defined process before the onset of clinical trials has to be strictly followed. Optimization of the target candidate, its pharmacokinetic and pharmacodynamic, absorption, distribution, metabolism, and excretion are mandatory before the development of a miRNA-based therapy. Furthermore, a fundamental step required as part of a pre-clinical evaluation is the toxicity testing, in order to ensure the drug safety and its possible applicability to humans. In addition to the off-target hybridization-dependent toxicity already discussed above, chemistry-related effects are a frequent cause of toxicity in oligonucleotide therapeutics, mainly associated to the dose regimen evaluation of an anti-miR drug. In fact, there is only a number of miRNAs that can be available for a specific anti-miR, therefore, adding further anti-miR will not help in the sequestration of more miRNAs and, on the other side, may become harmful when in excess. Knowing these parameters will be determinant in a correct design of dose regimen and be able to access a phase I clinical trial [72].

Currently, the diagnostic potential of miRNAs is developing by many biomedical companies, to define and commercialize biomarkers in specific fields (e.g., cardiovascular disease and cancer), in an attempt to identify disease-based miRNA profiles. Even though the miRNA therapeutic applicability can still be considered in its infancy, diagnostic progresses are definitely advancing, and a few miRNA panels are already available to clinicians.

## 7. Conclusions

OxS represents one of the most important mechanisms involved in the pathogenesis and progression of chronic degenerative diseases and their complications, suggesting that it may be considered an additional target for pharmacotherapy. In this context, the assessment of circulating OxS-related biomarkers may give several advantages, such as easy collection and assay bioavailability, retaining low cost, and the possibility of widespread use. However, none of these biomarkers has still reached a shared clinical application for many reasons. In fact, there are numerous aspects to be considered in the evaluation of oxidative stress, which require further evaluation and may affect its assessment and interpretation of results (Table 1).

First of all, the OxS status assessment is challenging, due to the extremely highly variable and dynamic nature of the pathophysiological mechanisms underlying oxidative stress. There is no biomarker in absolute that is more reliable than another; therefore, no OxS biomarker can be employed as a “gold standard”. Many other aspects regarding preanalytical (e.g., stability, interferences, handling and storage), analytical (e.g., assay/method choice, quality control), and postanalytic (e.g., reference ranges-at least cut-off) issues remain to be further evaluated.

Moreover, in the antioxidant supplementation planning, there is no shared agreement on the type of antioxidant, whether to use a single or a multi-marker approach, at which dosage, treatment time, etc. In the future, an attractive strategy would be the identification of key pathways/biomarkers that evidence patients that are in a state of OxS in a specific clinical context, allowing clinicians to profile these populations, that most likely benefit more from antioxidant supplementation [6]. The use of antioxidants in the treatment of cancer is at a more advanced stage than in cardiovascular and neurodegenerative contexts, and the first attempts are giving comforting results. Nonetheless, it is important to take into account the following determinant points: The antioxidant type and dosage, time of antioxidant supplementation/therapy, the background and status of the patient, and type of cancer and concomitant antitumoral therapy. Moreover, also sample sizes and used methodologies are of clinical relevance for result robustness.

Some evidence suggests that antioxidants may improve survival times, and increased tumor response, without interfering with chemotherapy [79]. Nonetheless, it will be hoped to develop soon an evidence-based approach to select the more appropriate antioxidants for the specific patient need. Conversely, the so-called “oxidation therapy”, which exploits the toxic properties of ROS, may be promising in specific experimental and clinical contexts. In particular, this approach implies a better knowledge of the molecular basis of OxS in different conditions, in order to develop targeted OxS pharmacological strategies related to specific pathways.

Many common drugs (e.g., statins) already widely used in a clinical setting, exert pleiotropic antioxidant effects. Moreover, there is an ongoing effort to identify new therapeutic modalities that specifically target some OxS processes, through utilization of nanotechnology-based drug delivery systems, gene therapies targeting overexpression of antioxidant systems, and anti-miRNAs strategies. Furthermore, in this exciting context, further research is needed to clarify the appropriate delivery and timing of these tools regarding their pharmacological properties.

Given the importance of OxS in the cardiovascular pathophysiology, reinforced by positive evidences in experimental studies, and despite numerous negative clinical trials about antioxidant interventions, available results should not hinder the pursuit in the field of OxS and antioxidant treatment, towards tailoring medical management to individual patient characteristics.

## Figures and Tables

**Table 1:** Main points to be considered in oxidative stress related assessment and result interpretation.

Steps in OxS Assesment	Main Points and Advices
***Biomarker selection***	select the most possible adequate analyte/s (single versus panel) for the population/setting investigated
	consider distribution volume/metabolism/clearance of the biomarker/s
***Test selection***	select the best assay/method for the population/setting investigated
***Population***	select the appropriate population (general population, patients) according to:
	*clinical setting (screening, diagnosis, prognosis, monitoring, treatment)*
	*athophysiology (diseases risk, diagnosis, stage)*
***Sample collection***	select biological sample (e.g., blood, urine, saliva)
	select anticoagulant and addition of stabilizers
	consider subject posture
	consider circadian rhythm (withdrawal time)
	fasting status
***Sample transport and processin***	sample handling (prompt transport, temperature, time)
	centrifugation modalities (rpm, temperature)
	prompt aliquot preparation for tests non immediately assayed
***Sample storage***	at −20 °C, best −80 °C
	avoid freeze-thaw cycles
	consider possible sample alterations with long storage time
***Sample testing***	evaluate additional steps (e.g., deproteinization, extraction/derivatization)
	assay specificity/sensibility
	evaluate presence of hemolysis, high lipid content
	consider assay/method agreement
	select “one spot” versus serial assessment
***Result interpretation***	availability of reference values/cut-off
	knowledge of assay/method limitations
	knowledge of variability due to additive determinants (e.g., genetic, physiological factors, lifestyle, intra/inter variability)
	awareness of different measurement units that can complicate result interpretation
***Antioxidant supplementation***	antioxidant dose
	antioxidant type
	single *versus* multi-antioxidant approach
	supplementation time
	interaction/synergism between antioxidant
	interference of dietary antioxidants
	higher requirement of vitamin intake (e.g., smokers, inactive people, etc)
	supplementation able to give a sufficient blood concentration to be effective
	avoid too high concentration, to exclude possible pro-oxidant effects
	initiation time according to the stage of disease (is antioxidant supplementation more effective to reverse mild damage?)
	redox homeostasis as target of supplementation
	selection of subjects/patients with increased oxidative stress to be supplemented
***Antioxidant drugs***	drugs that selectively target oxidative stress pathways increasing ROS production and cancer cellular death
	common drugs with antioxidant properties (e.g., statins, B-blockers, ACE-inhibitors, ARB in the cardiovascular field)
	also potentially effective for other disease prevention/treatment
	epigenetic miRNA-based approaches
